# MMPphg from the thermophilic *Meiothermus* bacteriophage MMP17 as a potential antimicrobial agent against both Gram-negative and Gram-positive bacteria

**DOI:** 10.1186/s12985-020-01403-0

**Published:** 2020-08-25

**Authors:** Feng Wang, Yan Xiong, Yao Xiao, Jian Han, Xianyu Deng, Lianbing Lin

**Affiliations:** 1 Faculty of Life Science and Technology, Kunming University of Science and Technology, 727 South Jingming Road, Kunming, 650500 Yunnan Province China; 2 Engineering Research Center for Replacement Technology of Feed Antibiotics of Yunnan College, 727 South Jingming Road, Kunming, 650500 Yunnan Province China

**Keywords:** *Meiothermus* bacteriophage, Genome sequencing, Endolysin, Antibiotic-resistant bacteria, Antimicrobial activity

## Abstract

**Background:**

New strategies are urgently needed to deal with the growing problem of multidrug-resistant bacterial pathogens. As the natural viruses against bacteria, recently, bacteriophages have received particular attention. Here, we identified and characterized a novel peptidoglycan hydrolase named MMPphg by decoding the complete genome sequence of *Meiothermus* bacteriophage MMP17, which was isolated in Tengchong hot spring in China and contains a circular genome of 33,172 bp in size and a GC content of 63.4%.

**Findings:**

We cloned the *MMPphg* gene, overproduced and purified the phage lytic protein, which contains a highly conserved M23 metallopeptidase domain and can be activated by Mg^2+^ and Zn^2+^. MMPphg is capable of withstanding temperatures up to 70 °C, and preserved more than 80% of its activity after a 30 min treatment between 35 and 65 °C. More interestingly, by disrupting bacterial cells, MMPphg exhibits surprising antimicrobial activity against both Gram-negative and Gram-positive pathogenic bacteria, especially antibiotic-resistant strains such as *Escherichia coli* O157, *Staphylococcus aureus* and *Klebsiella pneumonia*.

**Conclusions:**

In the current age of mounting antibiotic resistance, these results suggest the great potential of MMPphg, the gene product of bacteriophage MMP17, in combating bacterial infections and shed light on bacteriophage-based strategies to develop alternatives to conventional antibiotics for human or veterinary applications.

## Main text

With the uncontrolled and inappropriate use of antibiotics, multidrug-resistant bacterial pathogens are becoming increasingly common, which is one of the major concerns in combating serious bacterial infections, presenting great challenge for clinical therapy [1, 2]. To deal with this problem of antibiotic resistance, there is an urgent need to develop alternative therapeutic methods to replace or supplement antibiotics [3].

Bacteriophages (also known as phages) are natural viruses of microbes that specifically infect only host bacterial cells without affecting other microflora and exist in nearly every environment [4, 5]. They were found to be natural agents against bacteria in the early part of the twentieth century [6]. Recently, phage lytic enzymes that can destroy the cell wall of bacteria and thus cause host cell lysis have also gained new ground as alternative antibacterial agents because of their safety and high efficiency [7, 8].

*Meiothermus* bacteria belong to the family *Thermaceae* of the phylum *Deinococcus-Thermus*, which are moderately thermophilic strains and grow at temperatures of 35–68 °C [9]. Phages infecting *Meiothermus* bacteria are particularly interesting because they are sources of valuable agents resistant to denaturation at high temperatures. So far, however, studies on *Meiothermus* phages and their genome are scarce. Previously, we have isolated a *Meiothermus* phage named MMP17 from Tengchong hot spring in Yunnan Province of China [10]. In this study, we further decoded its complete genome sequence, and present the antimicrobial potential of MMPphg, an interesting gene product of *Meiothermus* phage MMP17.

**Fig. 1:**
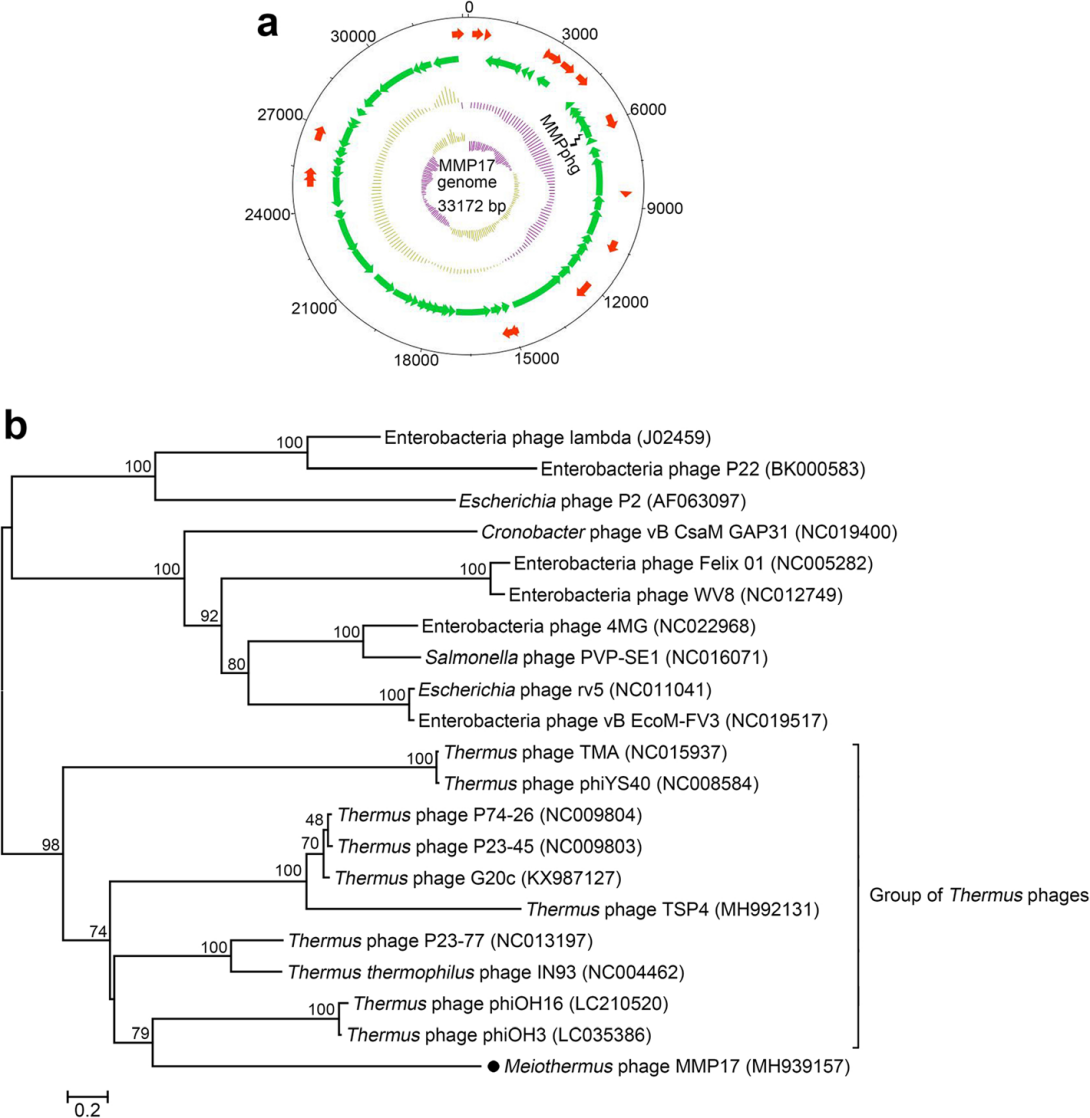
Genome map of phage MMP17 and whole-genome based neighbor-joining phylogenetic analysis. **a** Genome map of MMP17, from inside to outside: circle 1 demonstrates the GC skew [(G-C)/(G + C)]; circle 2 denotes the GC percentage plot; circles 3 and 4 show the ORFs on the minus strand (green) and plus strand (red) respectively; circle 5 displays the numbered scale with an interval of 3 kb. **b** Whole-genome-based phylogenetic tree of phage MMP17, which was created with MEGA6 program using the neighbor-joining method. The values at the nodes indicate bootstrap probabilities that were calculated based on 1000 replicates. Numbers in parentheses are the GenBank accession numbers of phage genomes

The genomic DNA of phage MMP17 was extracted using the phenol-chloroform method as previously described [11] and sequenced in the majorbio company (Shanghai, China) by Sanger sequencing. As shown in Fig. 1a, MMP17 has a small genome of 33,172 bp in size and a GC content of 63.4%; the high GC content of phage MMP17 is consistent with its growth temperature (63 °C). Based on reads overlapping features in the assembly, MMP17 has a circular genome. To validate this, the restriction enzymes digestion of MMP17 genome DNA was performed. As expected, the NcoI and HindIII (both have a single cutting site in the genome) digestion results were in line with the circular topology of MMP17 genome (Additional file 1: Fig. S1). Similar to many other thermophilic phage genomes such as P23–77 (GenBank accession: NC_013197.1) and IN93 (GenBank accession: NC_004462.2), this circular organization contributes to genome stability and may help the survival of phage MMP17 under extreme environment. This genome project has been deposited in GenBank under the accession number MH939157.

The BLASTn analysis further indicated that, at the nucleotide level, MMP17 exhibits no significant similarity to publicly available phage genomes in the GenBank database, suggesting its novelty. The closest neighbor of MMP17 is *Thermus aquaticus* Y51MC23 (GenBank accession: NZ_CP010822.1), but with only 1% sequence coverage and 82.9% identity, followed by *Meiothermus ruber* DSM1279 (GenBank accession: CP005385.1), with 1% sequence coverage and 80.4% identity. Open reading frame (ORF) prediction and annotation of MMP17 genome were performed with ORF finder on NCBI website and further refined with BLASTp program. Through these analyses, we found 86 ORFs in the MMP17 genome but there is no tRNA or rRNA gene in it. Of the 86 protein-encoding genes, only 26.7% (23/86) were assigned predicted functions (Additional file 3: Table S1). Consistent with current taxonomic results [9, 12], whole-genome-based neighbor-joining phylogenetic analysis demonstrated that there is a close evolutionary relationship between *Meiothermus* phage MMP17 and representatives of *Thermus* phages (Fig. 1b). In the phylogenetic tree, the most intimate relatives of MMP17 are *Thermus* phage phiOH16 (GenBank accession: LC210520) and *Thermus* phage phiOH3 (GenBank accession: LC035386). However, comparisons at the protein level by employing CoreGenes 3.5 [13] showed that only 2/86 (2.3%) proteins of phage MMP17 are homologous to those of both phiOH16 and phiOH3. Together, these results suggest that MMP17 indeed represents a novel phage.

**Fig. 2:**
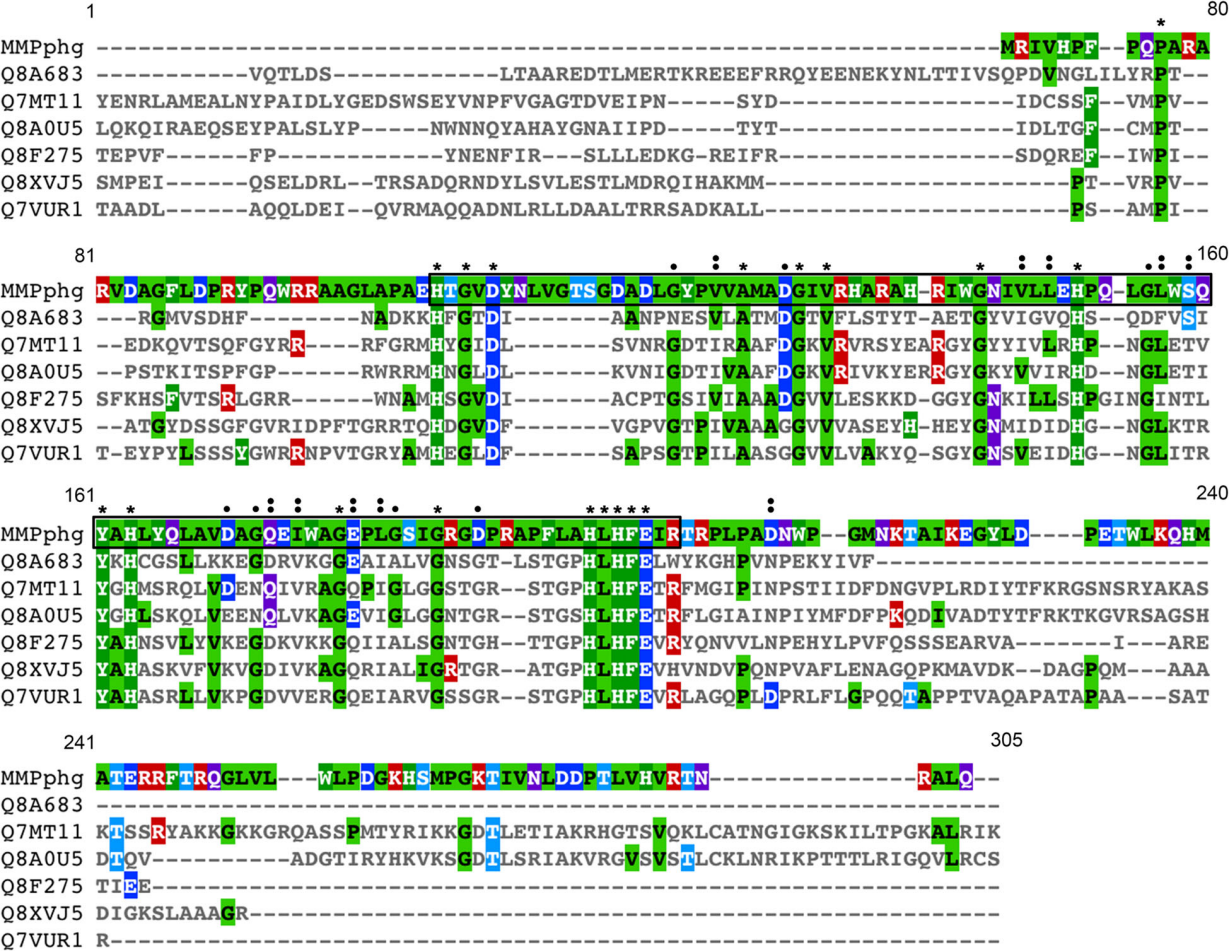
Multiple sequence alignment of MMPphg and other six representatives of M23 peptidase family. The alignment was carried out with CLUSTAL Omega (1.2.4). An asterisk (*) indicates positions that have a single, fully conserved residue, while colon (:) and period (.) denote conservation among groups of strong similarity and weak similarity, respectively. The amino acid residues of MMPphg within M23 peptidase domain were boxed. The UniProtKB/Swiss-Prot accession numbers Q8A683, Q7MT11, Q8A0U5, Q8F275, Q8XVJ5 and Q7VUR1 represent protein sequences of putative membrane peptidase from *Bacteroides thetaiotaomicron*, peptidase of M23/M37 family from *Porphyromonas gingivalis*, putative peptidase from *Bacteroides thetaiotaomicron*, peptidase of M23/M37 family from *Leptospira interrogans*, putative ipr002886 peptidase M23b family transmembrane protein from *Ralstonia solanacearum* and putative peptidase family M23/M37 protein from *Bordetella pertussis*, respectively. This figure was prepared with MView version 1.63

Among all the protein-encoding genes in MMP17 genome, ORF20 with a length of 633 bp is very interesting as it encodes a M23 family metallopeptidase which is composed of 210 aa (GenBank accession: QAY18044.1). The used initiation codon and termination codon of ORF20 are ATG and TAG, respectively. For convenience, we named ORF20 as *MMPphg* (peptidoglycan hydrolytic gene of MMP17) in this study. Next, we carried out the conserved domain analysis of MMPphg against NCBI Conserved Domain Database (CDD v3.17, last update: 2019-04-03) [14]. The results revealed that it contains a conserved M23 peptidase domain (NCBI domain architecture ID: 10480195) and is highly similar to peptidase of M23 family (accession: pfam01551) (Additional file 2: Fig. S2). Further, multiple sequence alignment of MMPphg and other six representatives of M23 peptidase family was performed by using Clustal Omega (version 1.2.4) with the default settings [15]. As shown in Fig. 2, there are 18 fully conserved residues (P77, H105, G107, D109, A127, G131, V133, G144, H151, Y161, H163, G178, G185, H196, L197, H198, F199 and E200) in the amino acid sequence of MMPphg. Interestingly, we also found a fully conserved motif HLHFE at the C-terminus of MMPphg M23 domain [16], which might be involved in the peptidoglycan-degrading process of bacterial cell wall and deserves further study.

**Fig. 3:**
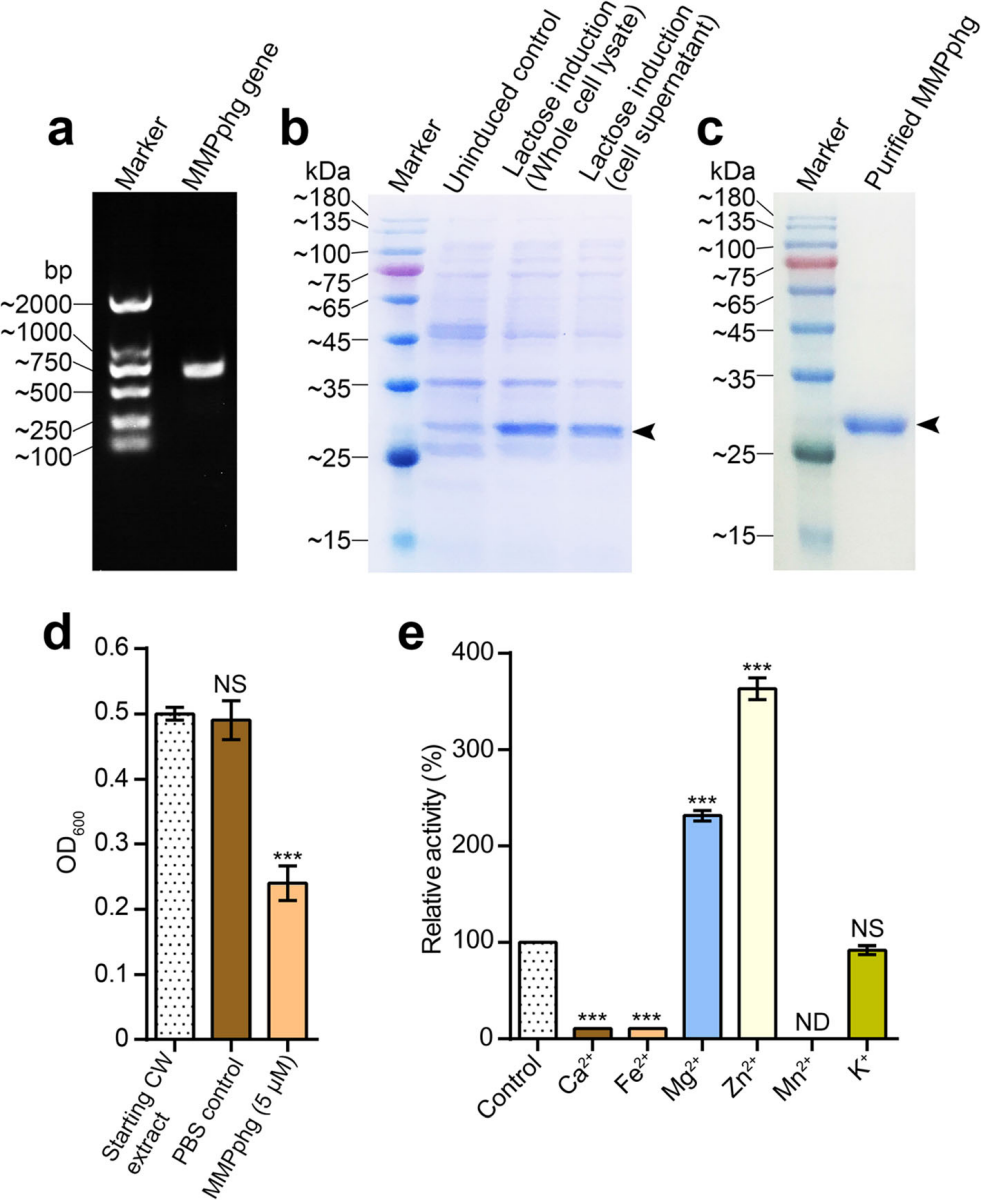
Gene cloning, protein expression and purification of MMPphg, the gene product of *Meiothermus* phage MMP17, and functional analysis of MMPphg as a metallopeptidase. **a** PCR amplification of *MMPphg* gene. **b** Lactose (1 g/L) was used for induction to overproduce MMPphg. **c** SDS-PAGE analysis of the purity of recombinant MMPphg, which is at approximately 26 kDa as the black arrowhead indicates. **d** MMPphg is able to digest cell wall (CW) extracted from *Meiothermus* sp. TG17, the host bacterium for phage MMP17. **e** Effects of metal ions on the lytic activity of MMPphg. Relative activity of MMPphg against *Meiothermus* sp. TG17 cells was calculated as percentage in relation to the non-treated control. Each experiment was repeated in triplicate; error bars indicate the standard deviation. *P* values were determined using the Student’s t test. ***, *P* < 0.001; ND, non-detectable; NS, not significant

Next, the DNA sequence of the putative peptidoglycan hydrolase, MMPphg, was cloned by PCR amplification (Fig. 3a). To overproduce MMPphg, *E. coli* Rosetta cells containing the recombinant expression vector pET28a-MMPphg were cultivated at 37 °C in LB medium supplemented with Kanamycin (50 μg/mL), and lactose (1 g/L) was used for induction of T7 promoter in the vector (Fig. 3b). The purification step was performed using HisTrap affinity column according to the manufacturer’s instructions (GE Healthcare, USA), and the purity of recombinant MMPphg was confirmed by 12% SDS-PAGE (Fig. 3c). The details of PCR primers, regular gene cloning, recombinant protein expression and purification are presented in Additional file 4. Subsequently, the capability of MMPphg to digest cell wall obtained from *Meiothermus* sp. TG17 (GenBank accession: GU329951), the host bacterium for phage MMP17, was confirmed (Fig. 3d). It is known that members of M23 peptidase family are usually zinc-dependent metallopeptidases [17], to confirm this, the effects of metal ions (Mn^2+^, Ca^2+^, Mg^2+^, Zn^2+^, Fe^2+^ and K^+^) on the lytic activity of MMPphg against *Meiothermus* sp. TG17 cells were evaluated. It can be seen from Fig. 3e that both Zn^2+^ and Mg^2+^ showed positive effects on MMPphg lytic activity. Especially, with a concentration of 1 mM, Zn^2+^ can reconstitute MMPphg activity to 363.4% compared with untreated control, possibly because of increased interaction of MMPphg with the peptidoglycan backbone mediated by divalent zinc cations [18]. Together, these results are in line with the function of MMPphg as a peptidoglycan hydrolase belonging to M23 peptidase family.

**Fig. 4:**
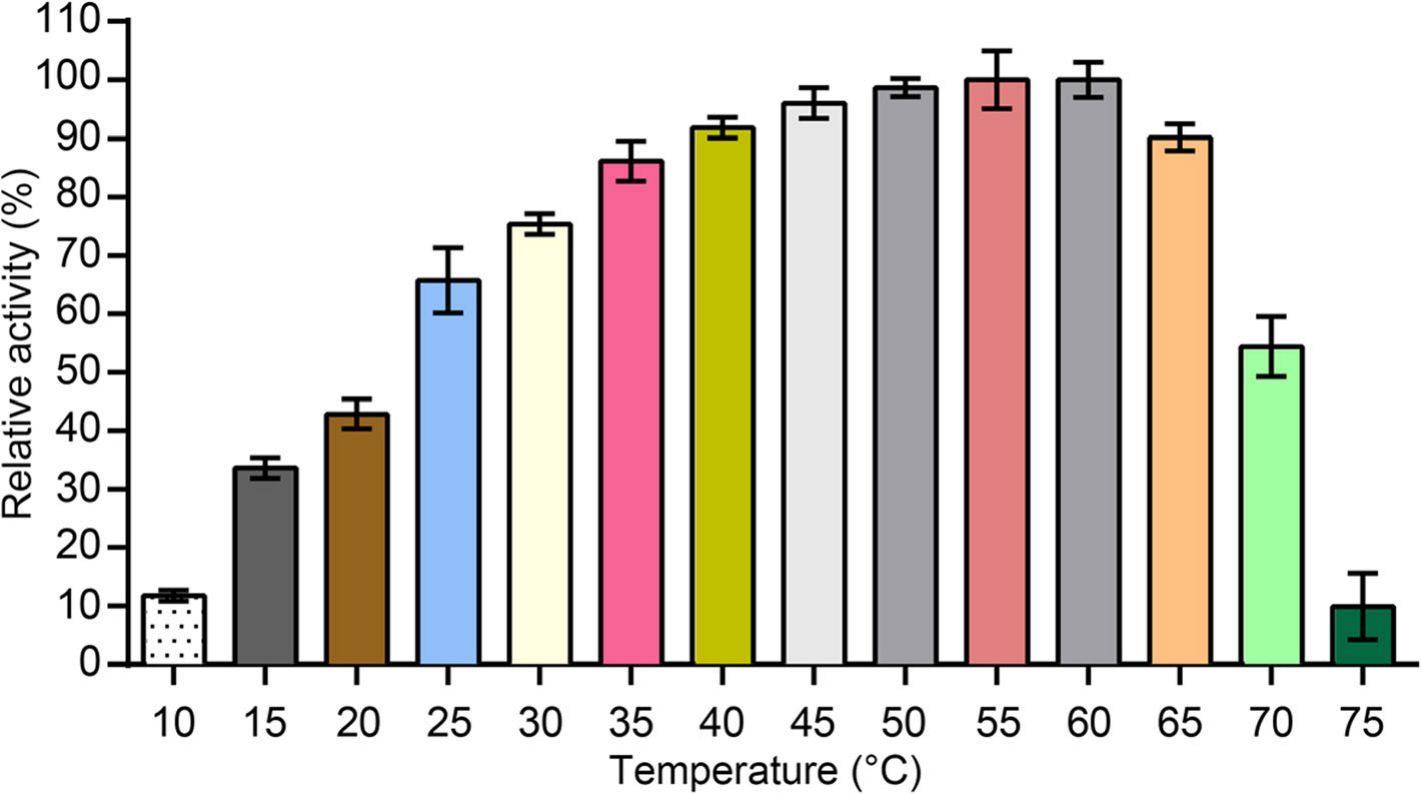
Thermostability of MMPphg, which was first incubated at different temperatures (from 10 to 75 °C) for 30 min, and then its activity was determined by the standard turbidity reduction assay against *Meiothermus* sp. TG17 cells. All data were normalized to the maximal lytic activity among the dataset (= 100%) and presented as mean ± standard deviation of *n* = 3 experiments

Furthermore, the thermostability of MMPphg was examined at different temperatures ranging from 10 to 75 °C. It is noteworthy that MMPphg retained more than 80% of its activity after a 30 min heat treatment between 35 and 65 °C, and still preserved more than 50% activity at 70 °C (Fig. 4), suggesting its considerable thermo-resistance to denaturation. Recently, a few lysins from thermophilic phages have been studied [19, 20]. For example, it was reported that an endolysin named PlyGVE2 from the deep-sea thermophilic *Geobacillus* phage GVE2 was relatively stable and active over a broad range of temperatures from 40 to 80 °C, and maintained 80% of its activity after a 30 min incubation at 55 °C [21, 22], which is similar to MMPphg. Moreover, the engineered chimeolysin of PlyGVE2, PlyGVE2CpCWB, could still preserve 57% activity after a treatment at 55 °C for 30 min [23]. More surprisingly, two endolysins with extremely high thermostability have also been reported [24, 25]. The first one is Ph2119 endolysin from the *Thermus scotoductus* MAT2119 bacteriophage Ph2119, and it retains approximately 86.7% of its initial activity after 6 h of incubation at 95 °C [24]. The other is Ts2631 endolysin from the *Thermus scotoductus* phage vB_Tsc2631. The Ts2631 endolysin has been demonstrated to be not only thermoresistant, maintaining 64.8% of its original activity after 2 h at 95 °C, but also highly thermodynamically stable, with a Tm of 99.8 °C [25]. By comparison, PlyC, an antimicrobial endolysin against *Streptococcus pyogenes*, completely lost its activity after a 50 °C heat treatment for 30 min [20, 26]. Therefore, we strongly believe that studies and development of these thermo-resistant phage proteins would be very helpful for biotechnological applications in humans or livestock.

**Fig. 5:**
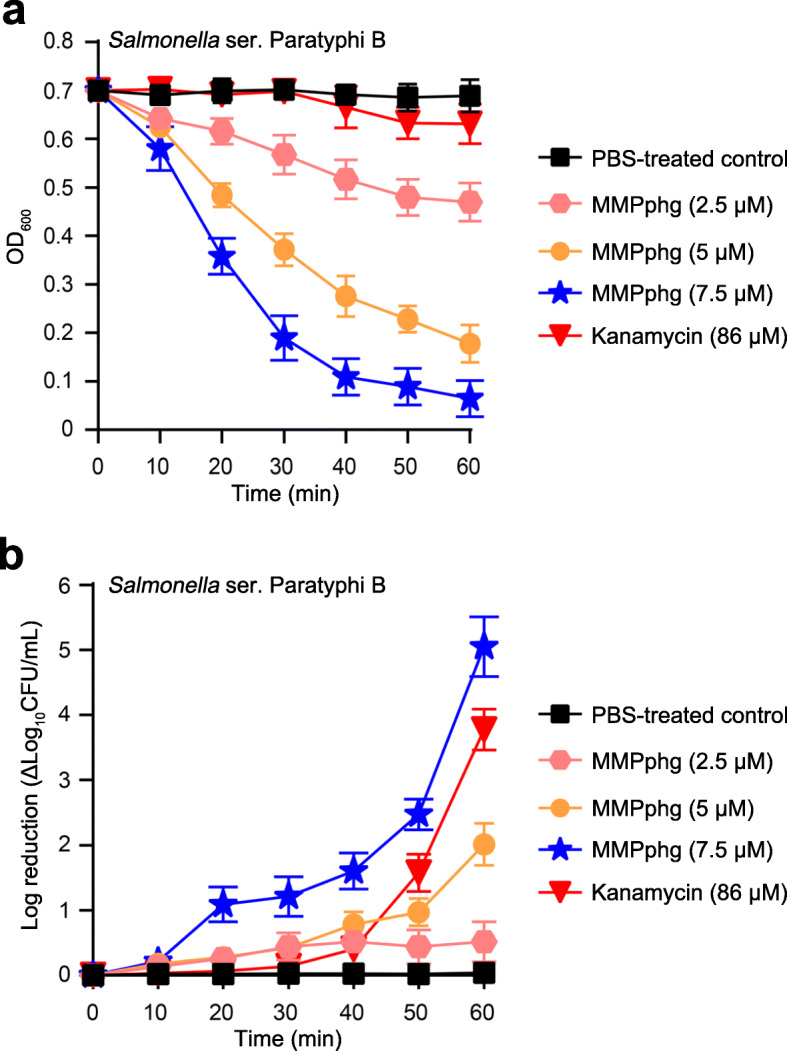
Dependence of MMPphg bacteriolytic and bactericidal activity on its concentration. **a** Turbidity reduction assays. Different concentrations of MMPphg were incubated with *Salmonella* ser*.* Paratyphi B at 37 °C, and the changes in OD_600_ were measured over time. **b** Bactericidal activity of MMPphg against *Salmonella* ser*.* Paratyphi B. After incubation at 37 °C over 60 min period (samples were taken at an interval of 10 min), aliquots were serially diluted and spread onto LB agar plates. Log kills were presented as ΔLog_10_ (CFU/mL) compared with bacterial counts before treatment. Kanamycin (50 μg/mL, approximately 86 μM) and PBS were used as the controls. Data were presented as mean ± SD, and all experiments were repeated in triplicate

Next, the bacteriolytic activity of MMPphg was tested at 37 °C in a turbidity reduction assay against *Salmonella* ser. Paratyphi B (without treatment by permeabilization agents)*,* which is a Gram-negative pathogenic bacterium that can contaminate many food products especially those of animal origin, and cause diseases in poultry and pork farming [27, 28]. As shown in Fig. 5a, the bacteriolytic activity of MMPphg increased proportionally to its concentration, and the curve pattern exhibited rapid kinetics of bacterial cell lysis by MMPphg. For example, with the MMPphg concentration of 7.5 μM, an OD_600_ reduction of 0.34 could be achieved after a 20 min incubation of MMPphg with the bacteria at 37 °C. For bacterial colony counting of *Salmonella* ser. Paratyphi B after MMPphg treatment on LB agar plates, the dose-dependent effect was also observed (Fig. 5b). Finally, a log_10_ reduction by 5.05 ± 0.46 in bacterial counts was reached upon exposure of 10^7^ CFU/mL of *Salmonella* ser*.* Paratyphi B cells to MMPphg (7.5 μM) at 37 °C for 1 h.

Considering the potential antimicrobial applications of MMPphg, we further tested its bactericidal activity against various Gram-negative or Gram-positive bacteria (see Additional file 4 for the detailed information of bacterial strains and methods). As shown in Table 1 and Additional file 4: Table S2, MMPphg exhibits antimicrobial activity against both Gram-negative and Gram-positive bacterial pathogens, which is quite rare and very interesting as currently most reported phage lysins only have lytic activity against Gram-negative or Gram-positive strains [3, 18, 29]. Recently, Plotka et al. have demonstrated that the Ts2631 endolysin, a promising antimicrobial agent from the extremophilic *Thermus scotoductus* bacteriophage vB_Tsc2631 [30], has a unique 20-residue N-terminus with 7 positively charged amino acids. And this 20-residue N-terminus of Ts2631 endolysin mediates peptidoglycan binding and is crucial for the antibacterial activity of the protein [31]. The same functional features were also highlighted in the case of the lytic enzyme LysC from *Clostridium intestinale* URNW [32], and it is noteworthy that the highly positively charged N-terminal tail of LysC is of vital importance for antimicrobial activity of the enzyme, which sheds light on new strategies to develop antimicrobial agents with superb activity. Accordingly, we found that the C-terminal tail of M23 peptidase domain of MMPphg (GRGDPRAPFLAHLHFEIRTR) contains six positively charged residues (underlined) as well as a highly conserved motif HLHFE. We hypothesize that this special 20-residue tail could assist penetration of MMPphg through the outer membrane of Gram-negative bacteria by electrostatic interactions with negatively charged molecules on the bacterial surface [33, 34], and hence be responsible for the observed antibacterial activity of MMPphg. Together, these clues suggest that MMPphg might be a multifunctional phage lytic protein with specific structure that is able to interfere with the outer membrane and hydrolyze bacterial peptidoglycan [18], which warrants future structure-based studies.

**Table 1:** Antimicrobial activity of MMPphg on various Gram-negative or Gram-positive bacteria. The significant log reduction units observed (≥ 1 log kill) are marked in bold

Strain	Antibiotic resistance ^a^	Antimicrobial activity (Log_10_ reduction) ^b^
*Escherichia coli*		
CMCC(B)44102	No	3.23 ± 0.15 (***)
*Staphylococcus aureus*		
ATCC6538	No	1.31 ± 0.03 (***)
KMUST1606BL1486	Yes	1.04 ± 0.11 (***)
*Salmonella* ser*.* Enteritidis		
CMCC(B)50335	No	1.08 ± 0.03 (***)
*Salmonella* ser. Typhi		
CGMCC1.1190	No	0.92 ± 0.01
*Salmonella* ser*.* Paratyphi B		
CMCC(B)50094	No	3.42 ± 0.15 (***)
*Shigella dysenteriae*		
KMUSTDS8	Yes	0.96 ± 0.01
KMUSTDS6	Yes	1.01 ± 0.01 (***)
*Klebsiella pneumoniae*		
13A14165	Yes	3.02 ± 0.01 (***)
13A14918	Yes	1.47 ± 0.03 (***)
13A15188	Yes	2.49 ± 0.07 (***)
13A15382	Yes	1.59 ± 0.03 (***)
1412SP0200	Yes	1.24 ± 0.02 (***)
1412SP0057	Yes	2.26 ± 0.10 (***)
13 V1837	Yes	0.97 ± 0.03
14 V0622	Yes	1.09 ± 0.02 (***)
1501SP0134	Yes	2.57 ± 0.07 (***)

^a^Detailed antibiotic-resistant information for the relevant strains is shown in Additional file 4: Table S2; ^b^Values represent mean ± standard deviation. For all tests, the antimicrobial activity was measured after incubating 10^6^ CFU/mL of the bacterial cells with 6 μM MMPphg at 37 °C for 1 h, and six independent replicates were run in each reaction. ***, *P* < 0.001 for MMPphg-treated cells versus PBS-treated controls

**Fig. 6:**
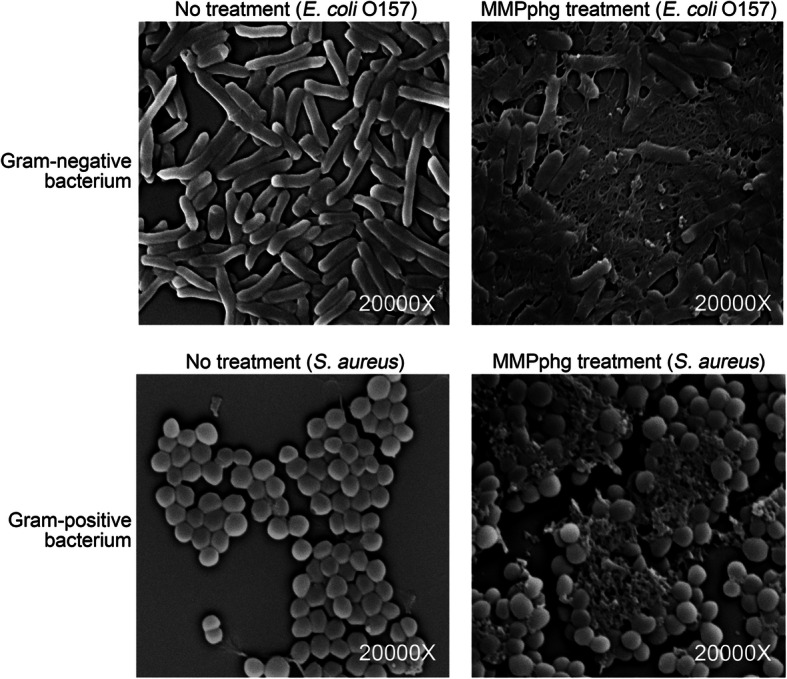
Scanning electron microscope showing the effects of exogenous MMPphg treatment on bacteria destruction of both Gram-negative (*E. coli* O157) and Gram-positive (*S. aureus*) cells. For all tests, 10^5^ CFU/mL of bacterial cells were treated with or without 5 μM MMPphg at 37 °C for 1 h. The magnification is 20,000 times the original size

In addition, we found that MMPphg also has broad antimicrobial activity against different multidrug-resistant strains of *Klebsiella pneumoniae* (Table 1); these Gram-negative strains belong to the ESKAPE group pathogens and are very difficult to treat in clinic care because of their antibiotic resistance and virulence [35]. To further investigate the detailed view of bacterial cell alteration after MMPphg treatment, scanning electron microscope (Quanta 200, FEI, Holland) analysis was performed according to the manufacturer’s instructions. As shown in Fig. 6, exogenous MMPphg treatment (5 μM) at 37 °C for 1 h could severely promote bacteria destruction of both Gram-negative (*E. coli* O157) and Gram-positive (*S. aureus*) cells, resulting in release of their intracellular components. These findings are consistent with the aforementioned activity of MMPphg and further confirmed the role of MMPphg as a phage-derived lytic enzyme with surprising antimicrobial activity against both Gram-negative and Gram-positive bacteria.

## Conclusions

Summarily, we report here the complete genome sequence of *Meiothermus* phage MMP17, and by annotating it, we identified and characterized a novel peptidoglycan hydrolase, MMPphg, which has antimicrobial activity against both Gram-negative and Gram-positive bacteria, especially multidrug-resistant pathogenic strains*.* In the current age of mounting antibiotic resistance, these results suggest the potential applications of MMPphg as an antimicrobial agent to fight against bacterial infections and provide insights into bacteriophage-based technologies to develop novel alternatives to antibiotics for human or veterinary applications. Still, future optimization and structure-based studies are needed to further increase the efficiency of MMPphg and delineate the underlying mechanism more precisely.

## Supplementary information

**Additional file 1: Fig. S1.** Patterns of restriction enzymes digestion of phage MMP17 genome DNA. **a** The positions of two restriction enzymes, NcoI and HindIII. Both have a single cutting site in the genome of phage MMP17. **b** Restriction enzymes cutting results of MMP17 genome DNA.

**Additional file 2: Fig. S2.** The conserved domain analysis of MMPphg at both nucleotide and protein levels.

**Additional file 3: Table S1.** Genetic features of open reading frames in the *Meiothermus* phage MMP17 genome. Protein sequences of the predicted ORFs of *Meiothermus* phage MMP17 were subjected to BLASTp program to analyze their best known matches on the NCBI website (https://blast.ncbi.nlm.nih.gov). The NCBI non-redundant database (nrdb) was used as the reference database, with the cutoff E-value set at 1E-05.

**Additional file 4: Table S2 and additional methods.** Table S2. Detailed antibiotic-resistant information for the bacteria used in this study.

## Data Availability

The complete genome sequence of *Meiothermus* bacteriophage MMP17 generated in this study was deposited in GenBank with the accession number MH939157.1 (https://www.ncbi.nlm.nih.gov/nuccore/MH939157.1/). All the other materials are available from the corresponding author on reasonable request.

## Abbreviations

MMPphg: Peptidoglycan hydrolytic gene of MMP17
MMP17: *Meiothermus Myoviridae* Phage 17
ORF: Open reading frame
SDS-PAGE: Sodium dodecyl sulfate polyacrylamide gel electrophoresis
ESKAPE: *Enterococcus faecium*, *Staphylococcus aureus*, *Klebsiella pneumoniae*, *Acinetobacter baumannii*, *Pseudomonas aeruginosa* and *Enterobacter* species
SEM: Scanning electron microscope
CFU: Colony-forming unit
PBS: Phosphate-buffered saline
LB: Luria Broth
CW: Cell wall
OD: Optical density
SD: Standard deviation
