# Supplementary figures and images for: MMPphg from the thermophilic Meiothermus bacteriophage MMP17 as a potential antimicrobial agent against both Gram-negative and Gram-positive bacteria
Source: Virol J. 2020 Aug 25;17:130. doi: 10.1186/s12985-020-01403-0 (PMC7448439; doi:10.1186/s12985-020-01403-0)

Figure S1

**a**

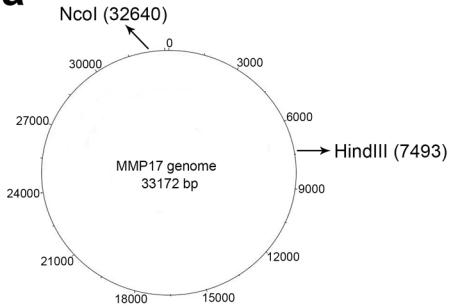

**b**

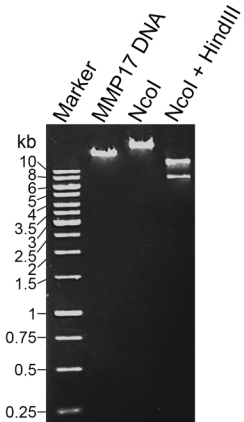

Supplement: Supplementary file 1 — Additional file 1: Fig. S1. Patterns of restriction enzymes digestion of phage MMP17 genome DNA. a The positions of two restriction enzymes, NcoI and HindIII. Both have a single cutting site in the genome of phage MMP17. b Restriction enzymes cutting results of MMP17 genome DNA. [file 12985_2020_1403_MOESM1_ESM.pdf]

Figure S2

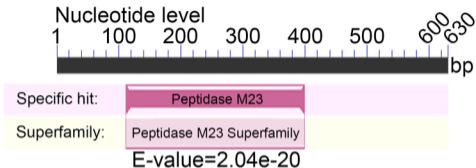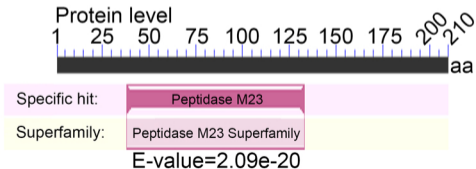

Supplement: Supplementary file 2 — Additional file 2: Fig. S2. The conserved domain analysis of MMPphg at both nucleotide and protein levels. [file 12985_2020_1403_MOESM2_ESM.pdf]
