# Supplementary material for: MMPphg from the thermophilic Meiothermus bacteriophage MMP17 as a potential antimicrobial agent against both Gram-negative and Gram-positive bacteria
Source: Virol J. 2020 Aug 25;17:130. doi: 10.1186/s12985-020-01403-0 (PMC7448439; doi:10.1186/s12985-020-01403-0)
Supplement: Supplementary file 3 — Additional file 3: Table S1. Genetic features of open reading frames in the Meiothermus phage MMP17 genome. Protein sequences of the predicted ORFs of Meiothermus phage MMP17 were subjected to BLASTp program to analyze their best known matches on the NCBI website (https://blast.ncbi.nlm.nih.gov). The NCBI non-redundant database (nrdb) was used as the reference database, with the cutoff E-value set at 1E-05. [file 12985_2020_1403_MOESM3_ESM.docx]

**Table S1.** Genetic features of open reading frames in the *Meiothermus* phage MMP17 genome.

| **ORF**  **name** | **Start** | **Stop** | **Strand** | **Gene length (bp)** | **Description** | **Best hit NCBI**  **accession ^a^** | **Identity**  **%** | **E-**  **value** |
| --- | --- | --- | --- | --- | --- | --- | --- | --- |
| ORF1 | 152 | 523 | + | 372 | Hypothetical protein | No hit | No hit | No hit |
| ORF2 | 587 | 790 | + | 204 | Hypothetical protein | No hit | No hit | No hit |
| ORF3 | 1315 | 701 | - | 615 | Hypothetical protein | No hit | No hit | No hit |
| ORF4 | 917 | 1231 | + | 315 | Hypothetical protein | No hit | No hit | No hit |
| ORF5 | 1925 | 1005 | - | 921 | Hypothetical protein | WP_110526098 | 50.77 | 1.03E-07 |
| ORF6 | 2163 | 1849 | - | 315 | MULTISPECIES: hypothetical protein | WP_110524630 | 55.88 | 2.42E-28 |
| ORF7 | 2437 | 2198 | - | 240 | Hypothetical protein | No hit | No hit | No hit |
| ORF8 | 2562 | 2440 | - | 123 | Hypothetical protein | No hit | No hit | No hit |
| ORF9 | 3053 | 2634 | - | 420 | Hypothetical protein | No hit | No hit | No hit |
| ORF10 | 2700 | 2933 | + | 234 | Hypothetical protein | No hit | No hit | No hit |
| ORF11 | 2860 | 3321 | + | 462 | Hypothetical protein | No hit | No hit | No hit |
| ORF12 | 3465 | 2902 | - | 564 | Hypothetical protein | No hit | No hit | No hit |
| ORF13 | 3368 | 3874 | + | 507 | Hypothetical protein | No hit | No hit | No hit |
| ORF14 | 4044 | 4493 | + | 450 | Helix-turn-helix domain-containing protein | WP_124973300 | 46.88 | 1.57E-07 |
| ORF15 | 4124 | 4459 | + | 336 | Hypothetical protein | No hit | No hit | No hit |
| ORF16 | 4715 | 4449 | - | 267 | Hypothetical protein DNA98_00965 | PZA08654 | 94.32 | 2.47E-52 |
| ORF17 | 5051 | 4752 | - | 300 | Hypothetical protein | No hit | No hit | No hit |
| ORF18 | 5224 | 4904 | - | 321 | Hypothetical protein | WP_135260860 | 73.63 | 2.66E-40 |
| ORF19 | 5735 | 5172 | - | 564 | Hypothetical protein | WP_126187738 | 57.81 | 2.33E-22 |
| ORF20 | 6211 | 5579 | - | 633 | M23 family metallopeptidase | WP_071677423 | 55.76 | 2.96E-79 |
| ORF21 | 5720 | 6154 | + | 435 | Hypothetical protein | No hit | No hit | No hit |
| ORF22 | 5734 | 6252 | + | 519 | Hypothetical protein | No hit | No hit | No hit |
| ORF23 | 6197 | 6547 | + | 351 | Hypothetical protein | No hit | No hit | No hit |
| ORF24 | 6421 | 6215 | - | 207 | Hypothetical protein | No hit | No hit | No hit |
| ORF25 | 7102 | 6629 | - | 474 | Hypothetical protein | WP_119340424 | 59.24 | 1.60E-60 |
| ORF26 | 8688 | 7111 | - | 1578 | Hypothetical protein | WP_119340425 | 88.36 | 3.28E-102 |
| ORF27 | 8548 | 8706 | + | 159 | Hypothetical protein | No hit | No hit | No hit |
| ORF28 | 9305 | 8697 | - | 609 | Hypothetical protein | WP_013014201 | 75.98 | 7.15E-108 |
| ORF29 | 8817 | 9170 | + | 354 | Hypothetical protein | No hit | No hit | No hit |
| ORF30 | 9813 | 9292 | - | 522 | Hypothetical protein | No hit | No hit | No hit |
| ORF31 | 10379 | 9306 | - | 1074 | Baseplate J protein | WP_119340404 | 64.33 | 1.93E-163 |
| ORF32 | 10261 | 10701 | + | 441 | Hypothetical protein | No hit | No hit | No hit |
| ORF33 | 10758 | 10381 | - | 378 | MULTISPECIES: hypothetical protein | WP_110527347 | 58.27 | 9.10E-41 |
| ORF34 | 11300 | 10761 | - | 540 | Hypothetical protein | WP_071677412 | 64.12 | 9.29E-67 |
| ORF35 | 11937 | 11272 | - | 666 | Hypothetical protein | WP_013014205 | 40.81 | 3.41E-41 |
| ORF36 | 12488 | 11934 | - | 555 | Hypothetical protein | WP_003047211 | 39.87 | 3.02E-32 |
| ORF37 | 11968 | 12573 | + | 606 | Hypothetical protein | No hit | No hit | No hit |
| ORF38 | 14731 | 12485 | - | 2247 | Tape measure domain protein | WP_013014207 | 34.24 | 2.55E-100 |
| ORF39 | 12500 | 13693 | + | 1194 | Hypothetical protein | No hit | No hit | No hit |
| ORF40 | 13152 | 13949 | + | 798 | Hypothetical protein | No hit | No hit | No hit |
| ORF41 | 13950 | 14261 | + | 312 | Hypothetical protein | No hit | No hit | No hit |
| ORF42 | 14295 | 14606 | + | 312 | Hypothetical protein | No hit | No hit | No hit |
| ORF43 | 14861 | 15166 | + | 306 | Hypothetical protein | No hit | No hit | No hit |
| ORF44 | 15196 | 14882 | - | 315 | Hypothetical protein | WP_027364023 | 31.58 | 1.28E-08 |
| ORF45 | 15084 | 15431 | + | 348 | Hypothetical protein | No hit | No hit | No hit |
| ORF46 | 15647 | 15204 | - | 444 | Hypothetical protein | WP_136365173 | 36.88 | 2.91E-20 |
| ORF47 | 17076 | 15658 | - | 1419 | phage tail sheath protein | ALJ90060 | 51.70 | 3.33E-155 |
| ORF48 | 16058 | 16372 | + | 315 | Hypothetical protein | No hit | No hit | No hit |
| ORF49 | 17270 | 17076 | - | 195 | Hypothetical protein | WP_071677406 | 59.02 | 2.52E-13 |
| ORF50 | 17719 | 17279 | - | 441 | Hypothetical protein | WP_071677405 | 40.41 | 3.31E-21 |
| ORF51 | 17927 | 17361 | - | 567 | Hypothetical protein | No hit | No hit | No hit |
| ORF52 | 18234 | 17716 | - | 519 | Putative tail completion protein | WP_013158346 | 49.13 | 5.24E-47 |
| ORF53 | 18335 | 17961 | - | 375 | Hypothetical protein | No hit | No hit | No hit |
| ORF54 | 18624 | 18235 | - | 390 | DUF1320 domain-containing protein | WP_003047195 | 54.62 | 1.46E-36 |
| ORF55 | 18842 | 18624 | - | 219 | Ribonucleotide-diphosphate reductase subunit | APD09743 | 53.33 | 1.99E-07 |
| ORF56 | 19713 | 18850 | - | 864 | Phage major capsid protein | WP_071677401 | 54.36 | 1.70E-109 |
| ORF57 | 20670 | 19723 | - | 948 | Mu-like prophage I protein | APD09741 | 79.31 | 9.29E-77 |
| ORF58 | 19856 | 20221 | + | 366 | Hypothetical protein | No hit | No hit | No hit |
| ORF59 | 22052 | 20856 | - | 1197 | Putative head morphogenesis protein | WP_119359365 | 57.14 | 2.00E-143 |
| ORF60 | 21321 | 21022 | - | 300 | Hypothetical protein | No hit | No hit | No hit |
| ORF61 | 23469 | 22006 | - | 1464 | DUF935 family protein | WP_003047184 | 63.88 | 0 |
| ORF62 | 22113 | 22502 | + | 390 | Hypothetical protein | No hit | No hit | No hit |
| ORF63 | 23051 | 22635 | - | 417 | Hypothetical protein | No hit | No hit | No hit |
| ORF64 | 23852 | 23445 | - | 408 | Hypothetical protein | WP_135260729 | 49.25 | 1.62E-28 |
| ORF65 | 23526 | 23795 | + | 270 | Hypothetical protein | No hit | No hit | No hit |
| ORF66 | 25223 | 23976 | - | 1248 | Hypothetical protein | No hit | No hit | No hit |
| ORF67 | 24802 | 24308 | - | 495 | Hypothetical protein | No hit | No hit | No hit |
| ORF68 | 24837 | 25283 | + | 447 | Hypothetical protein | WP_135260728 | 65.06 | 7.14E-165 |
| ORF69 | 25114 | 25512 | + | 399 | Hypothetical protein | No hit | No hit | No hit |
| ORF70 | 25695 | 25207 | - | 489 | Hypothetical protein | No hit | No hit | No hit |
| ORF71 | 26056 | 25688 | - | 369 | Hypothetical protein A0O31_01628 | APD09736 | 61.49 | 3.28E-50 |
| ORF72 | 26508 | 26062 | - | 447 | Hypothetical protein | No hit | No hit | No hit |
| ORF73 | 26486 | 27007 | + | 522 | Serine protease-like protein | MWR20480 | 69.73 | 2.66E-47 |
| ORF74 | 27388 | 26498 | - | 891 | Hypothetical protein | No hit | No hit | No hit |
| ORF75 | 27005 | 26649 | - | 357 | Hypothetical protein | No hit | No hit | No hit |
| ORF76 | 27503 | 27327 | - | 177 | DNA adenine methylase | TVT66069 | 63.40 | 1.91E-121 |
| ORF77 | 27772 | 27521 | - | 252 | Formate dehydrogenase-like protein | WP_013159817 | 87.04 | 6.63E-27 |
| ORF78 | 28278 | 27964 | - | 315 | Hypothetical protein | No hit | No hit | No hit |
| ORF79 | 29312 | 28431 | - | 882 | Hypothetical protein Mlute_00078 | RIH90156 | 91.35 | 2.63E-58 |
| ORF80 | 30903 | 29296 | - | 1608 | Hypothetical protein | No hit | No hit | No hit |
| ORF81 | 29638 | 30036 | + | 399 | Hypothetical protein | No hit | No hit | No hit |
| ORF82 | 31124 | 30900 | - | 225 | Hypothetical protein DBX97_01485 | PWM21870 | 33.09 | 1.97E-73 |
| ORF83 | 31687 | 31121 | - | 567 | Hypothetical protein | No hit | No hit | No hit |
| ORF84 | 32802 | 31759 | - | 1044 | Hypothetical protein TO73_0177 | ALJ90041 | 32.22 | 6.30E-20 |
| ORF85 | 32641 | 33045 | + | 405 | Bifunctional DNA primase/ polymerase | WP_049760435 | 44.41 | 1.09E-76 |
| ORF86 | 32805 | 472 | - | 32334 | Hypothetical protein | No hit | No hit | No hit |

^a^ Protein sequences of the predicted ORFs of *Meiothermus* phage MMP17 were subjected to BLASTp program to analyze their best known matches on the NCBI website (<https://blast.ncbi.nlm.nih.gov)>. The NCBI non-redundant database (nrdb) was used as the reference database, with the cutoff E-value set at 1E-05.
