# Supplementary material for: MMPphg from the thermophilic Meiothermus bacteriophage MMP17 as a potential antimicrobial agent against both Gram-negative and Gram-positive bacteria
Source: Virol J. 2020 Aug 25;17:130. doi: 10.1186/s12985-020-01403-0 (PMC7448439; doi:10.1186/s12985-020-01403-0)
Supplement: Supplementary file 4 — Additional file 4: Table S2 and additional methods. Table S2. Detailed antibiotic-resistant information for the bacteria used in this study. [file 12985_2020_1403_MOESM4_ESM.docx]

Table S2

Detailed antibiotic-resistant information for the bacteria used in this study.

| Strain | Antibiotic resistance |
| --- | --- |
| *Staphylococcus aureus* |  |
| KMUST1606BL1486 | Ciprofloxacin, clindamycin, erythromycin, gentamicin, levofloxacin, linezolid, moxifloxacin, nitrofurantoin, oxacillin, penicillin and rifampin |
| *Escherichia coli* O157 |  |
| KMUST401 | Streptomycin, tetracycline and ampicillin |
| *Klebsiella pneumoniae* |  |
| 13A14918 | Ceftriaxone, ampicillin, cefazolin, nitrofurantoin, amoxicillin and gentamicin |
| 13A14165 | Ampicillin, nitrofurantoin and cefoperazone |
| 13A15188 | Ampicillin and nitrofurantoin |
| 13A15382 | Ampicillin and nitrofurantoin |
| 1412SP0200 | Ampicillin, nitrofurantoin and ciprofloxacin |
| 1412SP0057 | Ceftriaxone, ampicillin, tazobactam, cefazolin, amitrazine, cefoxitin, amikacin, nitrofurantoin, amoxicillin and cefpiramide |
| 13V1837 | Ceftriaxone, ampicillin, cefazolin, aztreonam, nitrofurantoin and cefepime |
| 14V0622 | Ceftriaxone, ampicillin, cefazolin, aztreonam, cefoxitin and cefepime |
| 1501SP0134 | Ceftriaxone, ampicillin, cefazolin, amotrexone, nitrofurantoin, amoxicillin and cefpiramide |
| *Shigella dysenteriae* |  |
| KMUSTDS8 | Ofloxacin and sulfadiazine |
| KMUSTDS6 | Ofloxacin and sulfadiazine |

**Additional Methods**

**Bacterial strains, plasmids, and culture conditions**

*Staphylococcus aureus* cells were grown with shaking at 150 rpm in Nutrient Agar medium (10 g/L tryptone, 3 g/L beef extract, 5 g/L NaCl, pH = 7.2 ± 0.2) at 37 °C, and clinical isolates of *Klebsiella pneumoniae* were cultured at 37 °C in Brain Heart Infusion (BHI) medium (10 g/L tryptone, 12.5 g/L brain infusion powder, 5 g/L beef heart powder, 5 g/L NaCl, 2 g/L glucose, 2.5 g/L Na_2_HPO_4,_ pH = 7.4 ± 0.2) with shaking at 150 rpm. All the other bacterial strains used in this study were cultivated at 37 °C with shaking (150 rpm) in Luria Broth (LB) medium (10 g/L tryptone, 10 g/L yeast extract, 5 g/L NaCl, pH = 7.2 ± 0.2). The bacteria used as substrates for MMPphg were purchased from the American Type Culture Collection (*Staphylococcus aureus* ATCC6538) or China General Microbiological Culture Collection Center (*Salmonella* ser. Typhi CGMCC1.1190) or National Center for Medical Culture Collections [*Escherichia coli* CMCC(B)44102, *Salmonella* ser*.* Paratyphi B CMCC(B)50094 and *Salmonella* ser*.* Enteritidis CMCC(B)50335]. The different isolates of *Klebsiella pneumoniae* with their antibiotic-resistant patterns were kindly provided by Prof. Xueshan Xia and Dr. Yuzhu Song in the Research Center of Molecular Medicine of Yunnan Province, Kunming University of Science and Technology. All the other bacteria were stored in our laboratory. The record numbers and detailed antibiotic-resistant information of these strains are presented in Table 1 and Table S2. Plasmid pET-28a was purchased from Novagen (Madison, WI, USA). *Escherichia coli* strains Rosetta (for protein overproduction) and DH5a (for plasmid construction) were stored in our laboratory, and when necessary, the media were supplemented with kanamycin at 50 μg/mL or ampicillin at 100 μg/mL.

**Genome sequencing and bioinformatic analysis**

The genomic DNA of phage MMP17 was extracted using the phenol-chloroform method as previously described [[1](#_ENREF_1)], and sequenced in the majorbio company (Shanghai, China) by Sanger sequencing. Open reading frames (ORFs) were predicted with ORF finder on the NCBI website (https://www.ncbi.nlm.nih.gov/orffinder). All the ORF predictions were refined by BLASTp against NCBI nr database. The conserved domain analysis was carried out based on the NCBI Conserved Domain Database (CDD v3.17, last update: 2019-04-03) [[2](#_ENREF_2)]. To reveal the relationship between MMP17 and other phages, whole-genome based neighbor-joining phylogenetic tree was constructed using version 6.06 of Molecular Evolutionary Genetic Analysis (MEGA6) [[3](#_ENREF_3)]. Multiple sequence alignment of MMPphg and other six representatives of M23 peptidase family was carried out with CLUSTAL Omega (1.2.4) [[4](#_ENREF_4)]. This genome project has been deposited in GenBank under the accession number MH939157.1.

**Gene cloning, recombinant protein production, purification and validation of the capability of purified MMPphg to digest cell wall**

*MMPphg* gene was amplified by PCR program with gene-specific primers from the phage genome (forward: 5'-CATGCCATGGCAATGCGCATCGTTCATCCC-3' and reverse: 5'–ATTTGCGGCCGCTTGCAATGCGCGATTTG-3'); the 5' ends of forward and reverse primers were designed containing NcoI and NotI restriction sites (underlined), respectively, for directional cloning into the expression vector pET-28a. The PCR program was as follows: initial denaturation at 94 °C for 3 min, followed by 30 cycles of 94 °C for 45 s, 58 °C for 30 s, and 72 °C for 90 s. *E. coli* Rosetta containing the expression vector pET28a was then used as the host cells for recombinant protein expression. Lactose (1 g/L) was used for induction to overproduce the recombinant protein MMPphg. The purification was performed by using a HisTrap™ HP column according to the manufacturer's instructions (GE Healthcare, USA) and the protein supernatants were collected by eluting with 50-500 mM imidazole. The pooled proteins were subsequently dialyzed against 20 mM Tris-HCl (PH 7.4) and the final purity of recovered MMPphg was analyzed by 12% sodium dodecyl sulfate polyacrylamide gel electrophoresis (SDS-PAGE).

For analysis of cell wall digestion by purified recombinant MMPphg, the cell wall extract was generated from *Meiothermus* sp. TG17 (GenBank accession: GU329951), the host bacterium for phage MMP17, following the methods published previously [[5](#_ENREF_5), [6](#_ENREF_6)]. Briefly, the bacterial cells were cultured at 55 °C until an OD_600_ of 0.4-0.5 was reached as described previously [[7](#_ENREF_7)]. Then, they were collected by centrifuging at 1000 g for 10 min at room temperature, washed twice with phosphate-buffered saline (PBS) containing 137 mM NaCl, 2.7 mM KCl, 4.3 mM Na_2_HPO_4_, 1.4 mM KH_2_PO_4_, with a pH of 7.4. Subsequently, the cells were transferred into 4% (final concentration) SDS and boiled for 30 min, and the cell walls were then concentrated by centrifugation for 10 min at 30,000 g. The collected walls were further washed five times with PBS to remove remaining SDS. Finally, the prepared TG17 cell walls were used as the substrate and treated with 5 μM of MMPphg dissolved in PBS at 50 °C for 1 h with continuous stirring, and then the absorbance was assessed at 600 nm [[6](#_ENREF_6), [8](#_ENREF_8)].

**Measuring thermostability of MMPphg and effects of metal ions on its activity**

To investigate the features of MMPphg, *Meiothermus* sp. TG17 was used as the substrate and cultured at 55 °C until an OD_600_ of 0.4-0.5 was reached as described previously [[7](#_ENREF_7)]. Then, the bacterial cells were collected by centrifuging at 1000 g for 10 min at 4 °C, washed twice, and resuspended in PBS (137 mM NaCl, 2.7 mM KCl, 4.3 mM Na_2_HPO_4_, 1.4 mM KH_2_PO_4_, pH 7.4). For thermostability assays, MMPphg at 5 μM (128 μg/mL) dissolved in PBS was first heated at different temperatures (from 10 to 75 °C) for 30 min; subsequently, its activity was measured by the standard turbidity reduction assay at 50 °C in a 96-well microtiter plate (ShenYing Biotechnology, Haimeng, China). The lytic activity of MMPphg was determined by a decrease in OD_600_ in a plate reader (Wisdom Applied Science, mode 6500, Newark, DE, USA). Lysis of TG17 cells by MMPphg was also monitored without (negative control) or with the addition of metal ions (Mn^2+^, Ca^2+^, Mg^2+^, Zn^2+^, Fe^2+^ and K^+^) at a final concentration of 1 mM. Finally, the lytic activity of MMPphg was calculated at specific condition as follows: (OD_600_ (buffer only) – OD_600_ sample (MMPphg added))/initial OD_600_, as previously described [[9](#_ENREF_9), [10](#_ENREF_10)]. All assays were repeated in triplicate.

**Turbidity reduction assays and bacterial colony counting on LB agar plates**

To examine the antimicrobial activity of MMPphg, various Gram-positive or Gram-negative bacteria were grown and tested as described previously [[11](#_ENREF_11), [12](#_ENREF_12)]. Briefly, the bacteria were cultured at 37 °C in a rotary shaker at 150 rpm until an OD_600_ of 0.6-0.8 was reached; then they were centrifuged at 1000 g for 10 min at 4 °C, washed twice, and resuspended in PBS containing 137 mM NaCl, 2.7 mM KCl, 4.3 mM Na_2_HPO_4_, and 1.4 mM KH_2_PO_4,_ with a pH of 7.4. For turbidity reduction assays in a standard 96-well microtiter plate (ShenYing Biotechnology, Haimeng, China), the substrate bacteria, *Salmonella* ser. Paratyphi B, was used. After above-mentioned centrifugation, the bacterial cell pellets were suspended in PBS (with a starting value of OD_600_ being approximately 0.7; then they were treated with MMPphg dissolved in PBS at concentrations ranging from 2.5 to 7.5 μM in a final volume of 200 μL. Subsequently, the mixed samples were incubated at 37 °C over 60 min period (with an interval of 10 min) and the changes in OD_600_ were recorded over time in a plate reader (Wisdom Applied Science, mode 6500, Newark, DE, USA). To measure the antibacterial spectrum of MMPphg (see Table 1), approximately 10^6^ CFU/mL of bacterial cells were treated with 6 μM of MMPphg dissolved in PBS at 37 °C for 1 h, and the viable cell numbers and log kills were evaluated by plating on LB agar plates. The bactericidal activity of MMPphg was calculated as the relative inactivation in logarithmic units after the indicated time points as follows: Log_10_ (N_0_/N_i_), N_0_=number of untreated cells (in the negative control) and N_i_=number of treated cells counted after MMPphg incubation [[9](#_ENREF_9), [10](#_ENREF_10)]. The experiments were performed with at least three biological replicates.

**Scanning Electron Microscope (SEM) analysis**

The effects of exogenous MMPphg treatment on bacterial cell lysis were analyzed using scanning electron microscope (Quanta 200, FEI, Holland) according to the manufacturer's instructions. Briefly, the bacteria were collected and washed three times with PBS; then, 10^5^ of bacterial cells were incubated with 5 μM MMPphg (128 μg/mL) at 37 °C for 1 h. Subsequently, the bacterial lysates were fixed with 2.5% glutaraldehyde at 4 °C for 6 h and dehydrated in a graded ethanol series (30%, 50%, 60%, 70%, 80% and 90%) for 20 min each time. The samples were dried at room temperature for 24 h. Then the dried and frozen samples were used for SEM analysis as previously described [[13](#_ENREF_13)].

**Statistical analysis**

All data were expressed as mean ± standard deviation (SD). Statistical analysis was carried out in R software (version 3.2.2). P value < 0.05 was considered significant.

**Additional references**

1. Schmitz JE, Schuch R, Fischetti VA. Identifying active phage lysins through functional viral metagenomics. Appl Environ Microbiol. 2010;76:7181-7. https://doi.org/10.1128/aem.00732-10.

2. Marchler-Bauer A, Bo Y, Han L, He J, Lanczycki CJ, Lu S, et al. CDD/SPARCLE: Functional classification of proteins via subfamily domain architectures. Nucleic Acids Res. 2017;45:D200-D203. https://doi.org/10.1093/nar/gkw1129.

3. Tamura K, Stecher G, Peterson D, Filipski A, Kumar S. MEGA6: Molecular Evolutionary Genetics Analysis version 6.0. Mol Biol Evol. 2013;30:2725-9. https://doi.org/10.1093/molbev/mst197.

4. Sievers F, Higgins DG. Clustal Omega for making accurate alignments of many protein sequences. Protein Sci. 2018;27:135-45. https://doi.org/10.1002/pro.3290.

5. de Jonge BL, Chang YS, Gage D, Tomasz A. Peptidoglycan composition of a highly methicillin-resistant *Staphylococcus aureus* strain. The role of penicillin binding protein 2A. J Biol Chem. 1992;267:11248-54.

6. Lim J-A, Shin H, Heu S, Ryu S. Exogenous lytic activity of SPN9CC endolysin against Gram-negative bacteria. J Microbiol Biotechnol. 2014;24:803-11. https://doi.org/10.4014/jmb.1403.03035.

7. Lin L, Han J, Ji X, Hong W, Huang L, Wei Y. Isolation and characterization of a new bacteriophage MMP17 from *Meiothermus*. Extremophiles. 2011;15:253-8. https://doi.org/10.1007/s00792-010-0354-z.

8. Grabowska M, Jagielska E, Czapinska H, Bochtler M, Sabala I. High resolution structure of an M23 peptidase with a substrate analogue. Sci Rep. 2015;5:14833. https://doi.org/10.1038/srep14833.

9. Plotka M, Kaczorowska AK, Morzywolek A, Makowska J, Kozlowski LP, Thorisdottir A, et al. Biochemical characterization and validation of a catalytic site of a highly thermostable Ts2631 endolysin from the *Thermus scotoductus* phage vB_Tsc2631. PLoS One. 2015;10:E0137374. https://doi.org/10.1371/journal.pone.0137374.

10. Plotka M, Kapusta M, Dorawa S, Kaczorowska AK, Kaczorowski T. Ts2631 endolysin from the extremophilic *Thermus scotoductus* bacteriophage vB_Tsc2631 as an antimicrobial agent against Gram-negative multidrug-resistant bacteria. Viruses. 2019;11:E657. https://doi.org/10.3390/v11070657.

11. Yang H, Wang M, Yu J, Wei H. Antibacterial activity of a novel peptide-modified lysin against *Acinetobacter baumannii* and *Pseudomonas aeruginosa*. Front Microbiol. 2015;6:1471. https://doi.org/10.3389/fmicb.2015.01471.

12. Wang F, Ji X, Li Q, Zhang G, Peng J, Hai J, et al. TSPphg lysin from the extremophilic *Thermus* bacteriophage TSP4 as a potential antimicrobial agent against both Gram-negative and Gram-positive pathogenic bacteria. Viruses. 2020;12:E192. https://doi.org/10.3390/v12020192.

13. Cheng M, Zhang L, Zhang H, Li X, Wang Y, Xia F, et al. An ointment consisting of the phage lysin LysGH15 and apigenin for decolonization of methicillin-resistant *Staphylococcus aureus* from skin wounds. Viruses. 2018;10:E244. https://doi.org/10.3390/v10050244.
